# Age-related variations in HbA1c improvements: insights from a telehealth-supported community-based intervention

**DOI:** 10.1093/geroni/igaf121

**Published:** 2025-10-30

**Authors:** Laura Porterfield, Xiaoying Yu, Amber B Amspoker, Craig A Johnston, Aanand D Naik, Salim S Virani, Christie M Ballantyne, Ashok Balasubramanyam, Elizabeth M Vaughan

**Affiliations:** Department of Family Medicine, University of Texas Medical Branch, Galveston, Texas, United States; Sealy Institute for Vaccine Sciences, University of Texas Medical Branch, Galveston, Texas, United States; Department of Biostatistics, University of Texas Medical Branch, Galveston, Texas, United States; Department of Medicine, Baylor College of Medicine, Houston, Texas, United States; Michael E. DeBakey VA Medical Center, Houston, Texas, United States; Department of Health and Human Performance, University of Houston, Houston, Texas, United States; Department of Management, Policy, and Community Health, University of Texas School of Public Health, Houston, Texas, United States; Section of Cardiology. Aga Khan University, Karachi, Pakistan; Department of Population Health, Aga Khan University, Nairobi, Kenya; Department of Medicine, Baylor College of Medicine, Houston, Texas, United States; Department of Medicine, Baylor College of Medicine, Houston, Texas, United States; Department of Medicine, Baylor College of Medicine, Houston, Texas, United States; Department of Internal Medicine, University of Texas Medical Branch, Galveston, Texas, United States

**Keywords:** Diabetes, Group education, Minority health, Community health workers, Telehealth

## Abstract

**Background and Objectives:**

We previously demonstrated that a telehealth-supported community health worker (CHW) intervention significantly improved clinical outcomes in diabetes care. However, the extent to which these benefits vary across different age groups remains unclear. This study evaluated the effectiveness of a CHW-led multidimensional diabetes intervention in reducing HbA1c across age groups.

**Research Design and Methods:**

We conducted a retrospective analysis of 10 studies (*n* = 301) that focused on low-income Hispanic adults with or at risk for type 2 diabetes. The intervention included CHW-participant coaching via mobile Health (mHealth), monthly CHW-led group education, and bidirectional mHealth feedback among participants, CHWs, and clinicians. Outcomes included HbA1c changes from baseline to 6 months and an analysis of CHW-participant conversation data.

**Results:**

HbA1c levels improved across all age groups from baseline to 6 months, with statistically significant reductions observed in individuals aged 40-66 years (*p* < .05). As age increased from 40 to 49 years (*n* = 78), HbA1c reductions ranged from −0.83% to −1.18% (*p* = .013 to *p* < .001). By age 50-65 years (*n* = 182), the trend reversed, with smaller improvements observed as age increased (−1.16% to −0.61%, *p* < .001 to *p* = .016). Beyond 65 years (*n* = 27), HbA1c changes plateaued and were not statistically significant. Medication-related concerns were more prevalent among adults ≥65 years (73.7%) compared to those <65 years (44.1%) (*p* = .014).

**Discussion and Implications:**

HbA1c improved across all ages except in older adults, suggesting that age may play a role in intervention effectiveness. Targeted strategies and further research are needed to understand and address these age-related differences.

Innovation and Translational Significance: This study highlights the age-dependent effectiveness of a telehealth-supported Community Health Worker intervention for managing type 2 diabetes in low-income Hispanic adults. While HbA1c reductions were significant across all age groups, the degree of improvement plateaued with increasing age. These findings suggest that older adults may experience less benefit from the intervention, potentially due to age-related factors not addressed by a general program. Tailored approaches that address the unique needs of older adults are essential to maximize intervention effectiveness and improve diabetes care outcomes across age groups.

## Introduction

The number of people living with diabetes has surged, with the global prevalence of diabetes quadrupling over the last 3 decades.^1^ As diabetes prevalence increases, the number of adults living with diabetes has also grown significantly. The Centers for Disease Control and Prevention estimates that 4.8% of younger adults (18-44 years), 18.9% of middle-aged adults (45-64 years), and 27.3% of older adults in the United States have diabetes.^2^ Tailoring treatment strategies to meet the specific risks and needs of different age groups is crucial for enhancing care. However, due to limited knowledge on how the effectiveness of various treatment methods varies throughout the lifespan, treatment approaches often remain uniform, failing to consider the distinct needs of each age group.^3^

The challenges in treating diabetes may vary across ages and life stages due to changing physiological, psychological, and social factors. Younger adults face an increased lifetime risk for complications and disability due to longer disease exposure from earlier onset, yet they may also be undertreated compared to other adult age groups.^4^^,^^5^ Additionally, younger adults encounter unique challenges in managing diabetes while navigating secondary education, early career establishment, the development of adult social networks, primary reproductive years, and early family life.^5-7^ For middle-aged adults, barriers include social commitments, schedule demands, and biophysical changes.^8^ Meanwhile, older adults face management challenges such as polypharmacy, higher comorbidity burdens, increased social barriers (eg, transportation barriers and social isolation), and age-related conditions such as cognitive impairment, decreased mobility, and frailty.^3^^,^^9^

Current diabetes care standards emphasize lifestyle and ­pharmacotherapy as key components of management.^10^ However, therapeutic inertia and challenges with sustained self-management limit the effectiveness of traditional models that primarily rely on in-person visits.^11^ Emerging evidence highlights the need for multidisciplinary, team-based approaches that incorporate telehealth and group interventions.^11-14^ Community Health Workers (CHWs), trusted community members who connect patients with healthcare and social services, have been shown to be cost-effective in improving glycemic control and reducing healthcare utilization.^15^^,^^16^ Group-based diabetes education interventions also outperform usual care in enhancing glycemic control and promoting lifestyle changes.^16^^,^^17^ Additionally, telehealth and mobile health (mHealth) interventions complement traditional care by improving self-management.^11^^,^^18^

Our prior studies have demonstrated the effectiveness of CHW-led mHealth and group education interventions in improving glycemic control.^19-21^ However, there is limited understanding of the effectiveness of these interventions across various age groups. To address this gap, we conducted a retrospective analysis of ten CHW-led studies involving Hispanic patients with or at risk for type 2 diabetes. The primary aim of this study was to assess the effectiveness of these interventions in changing HbA1c across different age groups. We hypothesized that the impact of CHW-led mHealth and group education interventions on HbA1c would vary by age across the 10 studies.

## Methods

### Study design and participants

This was a retrospective analysis of our CHW-led diabetes models from 2015 to 2024. Detailed methodology and outcome improvements from these interventions are described elsewhere.^20-27^ The intervention included weekly to bimonthly CHW-participant coaching and monthly group education sessions, where CHWs provided education and personalized coaching on diabetes self-management. CHWs facilitated ­communication of patient concerns through a closed-loop ­feedback system between CHWs and clinician champions.^20^ CHWs documented each participant interaction on a secure, HIPAA-compliant spreadsheet platform. The studies received approval from the Institutional Review Board, and all participants provided informed consent.

Eligible participants were Spanish-speaking Hispanic adults with HbA1c ≥6.5% or at risk (HbA1c 5.7%-6.4%) for type 2 diabetes.^11^ Individuals were excluded if they were pregnant, had type 1 diabetes, or were on medications that could alter HbA1c, such as corticosteroid therapy. Participants received care in community clinics serving uninsured individuals with incomes below 150% of the federal poverty level, with more than 50% of participants being undocumented.

### Measures

We utilized an intention-to-treat analysis to assess the continuous trends in changes in HbA1c from baseline to 6 months by age, as all participants completed at least a 6-month program. To ensure completeness, we also examined trends in HbA1c by age from baseline to the end of the study for participants in 6-month, 12-month, and 15-month programs (eg, baseline to 12 months for a participant in a 12-month program). Clinical outcome values were defined as the target timepoint ± 1 month.

We also reviewed documented data from CHW–participant conversations in a subset of 5 studies (*n* = 223) to explore diabetes-related concerns reported by individuals of different ages. These concerns were open-ended, participant-reported issues recorded on the team spreadsheet. Due to the high prevalence of medication-related concerns, defined as refills, dosing questions, or side effects, we conducted a subsequent analysis to examine differences by age. We grouped participants by age, comparing those older than 50 with those younger than 50, as 50 is often considered a pivotal point for health and physical performance.^28^ We also compared older adults (defined as ≥65 years) with the rest of the population.^29^ This submission includes content edited using ChatGPT (version GPT-4) by OpenAI. The AI was used to assist in editing and refining the text based on user-provided drafts and instructions.

### Statistical analysis

Analyses were performed using SAS 9.4 (SAS Institute, Cary, NC).^30^ Clinical significance was defined as an HbA1c reduction of >0.5%, while statistical significance was set at *p* < .05. Data were summarized using descriptive statistics (eg, standard deviations for continuous variables and frequencies and proportions for categorical variables). Binomial data from CHW–participant conversations for all ages were analyzed using a series of chi-square tests. We used a linear mixed model, with HbA1c at baseline and month 6 as the outcome. For study endpoint data, if unavailable within the ± month window, the 3-month value was used. If two 3-month values were available (before and after the target), they were averaged. If neither was available, the data were considered missing. The mixed model assumes the missingness is random based on observed data and includes both random and fixed effects. The random intercept effect was included to account for varying baseline HbA1c values among individuals and the correlations among repeated measured HbA1c values (missing 6-month HbA1c values: *n* = 33) from the same participant. The fixed effects included age, month (baseline and 6 months), and the age-by-month interaction. Importantly, we included the interaction term to evaluate how changes in HbA1c from baseline to 6 months vary by age.

Instead of using the linear term for age, which assumes the age effect is monotone with a constant slope, we adopted a restricted cubic spline that allows for a non-monotone effect (eg, a convex curve).^31^^,^^32^ This method splits the range of values for age, with “knots” defining the end of one segment and the start of the next. For knot placement, we adopted clinically meaningful cutoffs (age 40, 50, 55, and 65), which are close to the data-driven cut-offs (40, 50, 57, and 67, corresponding to the 5th, 35th, 65th, and 95th percentiles, respectively).^31-33^ Separate curves (polynomials of degree 3) are fit to each segment, except for the 2 tails, which are constrained to be linear to avoid poor behavior at the extremes and reduce the number of parameters that need to be estimated.

To determine the number and location of the knots, we first used a nonparametric technique, locally estimated scatterplot smoothing,^32^^,^^34^ a graphical diagnostic of trends, to explore the nonlinear relationship between observed HbA1c changes and age and the locations for turns of the changes.^31-33^ The placement of knots is not as important as the selection of the number of knots for the restricted cubic splines. We then chose 4 knots, per recommendation, based on the available sample size to ensure a good balance between model fit and loss of precision of the model, as well as to maintain the parsimony of the model (8 parameters for the final spline model for the fixed effects, given a total of 568 observations from 301 participants).^31-33^

Due to the small sample sizes for ages <40 (14 participants, 25 observations) and >75 (5 participants, 8 observations), we focused on estimates for participants aged 40-75 years. To visualize age-specific changes, we estimated the change in HbA1c and the 95% confidence interval for each age (from 40 to 75) using this model, considering the limited data at the extremes. A step-down Bonferroni method was applied to adjust p-values for multiple comparisons.

## Results

Individual participant (*n* = 301) demographics and baseline characteristics are summarized in Table 1. Many participants were female (65.4%). The mean age was 53.4 years. At baseline, participants’ body mass index averaged 32.9 kg/m^2^, blood pressure was 131/78 mmHg, and HbA1c was 8.2%. More than half of participants (52%) participated in a 6-month program. The most common type of diabetes therapy was oral medications only (50.8%), followed by injectable and oral therapy (14%), and lifestyle only (11%).

**Table 1.** Baseline demographics and characteristics of individual participants (*n *= 301).

Variable	*n* (%)	Mean (*SD*)
**Age (years)**		
** <30**	3 (1.0)	
** 30-39**	11 (3.7)	
** 40-49**	78 (26.0)	
** 50-59**	144 (48.0)	
** 60-64**	38 (12.7)	
** 65-74**	20 (6.6)	
** 75+**	7 (2.3)	
**Sex**		
** Female**	197 (65.4)	
** Male**	104 (34.6)	
**Ethnicity**		
** Hispanic**	301 (100.0)	
**Diabetes category**		
** At-risk for diabetes^a^**	33 (10.7)	
** Diabetes^b^**	268 (89.3)	
**Diabetes therapy (*n* = 231)**		
** Lifestyle modifications only**	33 (14.3)	
** Oral medications only**	153 (66.2)	
** Injectables only**	3 (0.9)	
** Oral + Injectable medications**	42 (18.2)	
**Age (years)**		53.4 (8.8)
**Disease control**		
** HbA1c (%)**		8.2 (2.3)
** Systolic blood pressure (mmHg)**		130.8 (18.7)
** Diastolic blood pressure (mmHg)**		78.0 (9.3)
** Body mass index (kg/m^2^)**		32.9 (6.7)
**Program duration**		
** 6 mo**	156 (52.0)	
** 12 mo**	78 (26.0)	
** 15 mo**	67 (22.0)	

a Elevated HbA1c (5.7%-6.4%), fasting glucose (100-125 mg/dL), and/or overweight or obese (BMI ≥ 25.0 kg/m^2^).

b HbA1c ≥6.5%.

Characteristics of the 10 included studies are described in Table 2. The studies consisted of 7 randomized clinical trials (RCTs) and 3 cohort studies. The studies varied in duration, with 7 lasting 6 months, 2 lasting 12 months, and 1 lasting 15 months. Consistent with the clinics’ population, most participants were female (43%-91%), and the average age was in the 50s.

**Table 2.** Characteristics of studies (*n* = 10) evaluating diabetes programs in the community, totaling 301 participants.

Study	Design	Intervention (*n*)	Female (%)	Mean age (years) (*SD*)	Study duration (months)	Time period (month/year)
**1**	RCT	25	83	51.9 (8.3)	6	9/2015-2/2016
**2**	RCT	22	91	59.1 (7.5)	6	1/2018 6/2018
**3**	RCT	22	64	52.8 (5.1)	6	7/2018-12/2018
**4**	Cohort	14	86	55.4 (10.4)	6	7/2018-12/2018
**5**	Cohort	33	62	53.3 (6.8)	6	1/2019-7/2019
**6**	RCT	22	57	52.5 (7.7)	6	9/2019-2/2020
**7**	Cohort	18	47	58.8 (10.7)	6	6/2020-11/2020
**8**	RCT	67	67	52.3 (10.3)	15	4/2021-6/2022
**9**	RCT	29	43	52.1 (7.1)	12	9/2022-8/2023
**10**	RCT	49	51	52.7 (9.7)	12	9/2023-8/2024

RCT = randomized clinical trial.

**Figure 1.**
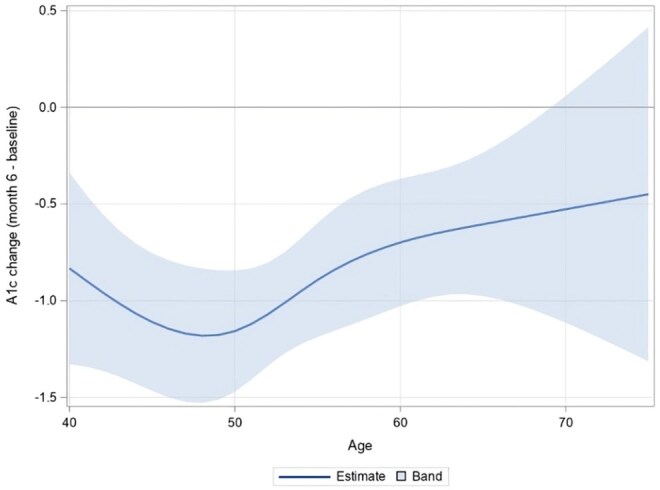
Estimated age-specific HbA1c changes baseline to 6 months, with 95% confidence intervals.

Figure 1 shows the average change in HbA1c from baseline to 6 months by age, with Supplementary Table 1 detailing the HbA1c changes for each age along with the 95% confidence interval. HbA1c levels improved from baseline to 6 months across all ages, with statistically significant reductions observed in individuals aged 40-66 years (all *p*-values < .05). As age increased from 40 to 49 years, HbA1c reductions moved from −0.83% (*p* = .013) to −1.18% (*p* < .001). In contrast, from ages 50 to 66 years, the reductions were smaller, ranging from −1.16% (*p* < .001) to −0.61% (*p* = .016). Beyond 66 years, HbA1c changes plateaued and were not statistically significant.

**Figure 2.**
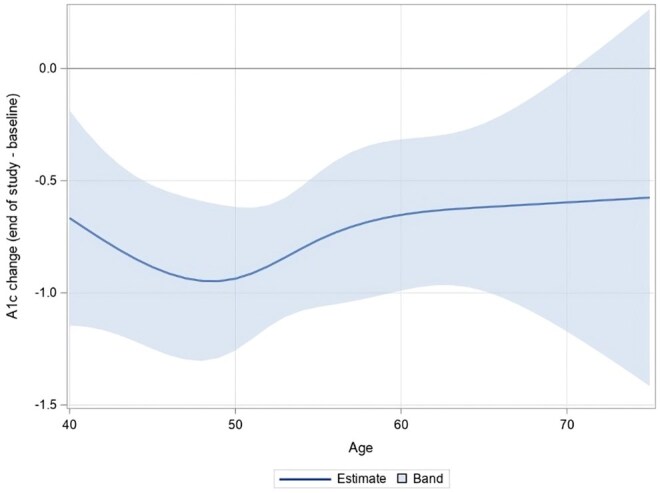
Estimated age-specific HbA1c changes from baseline to study endpoint (6, 12, or 15 months), with 95% confidence intervals.

For completeness, we also evaluated changes in HbA1c from baseline to the participants’ study endpoint by age (Figure 2), with Supplementary Table 2 providing the HbA1c changes for each age along with the 95% confidence intervals. HbA1c levels improved across all ages from baseline to 6 months, with statistically significant reductions observed in individuals aged 41-66 years (all *p* values < .05). Once again, the largest reductions were observed in those aged 40 (−0.67%, *p* = .07) to 49 years (−0.95%, *p* < .001). From ages 50 (−0.94%, *p* < .001) to 62 (−0.63%, *p* = .004) years, the trend reversed, with smaller improvements as age increased. Beyond age 67, HbA1c changes plateaued and were not statistically significant.

Descriptive exploratory data of CHW-participant conversations for 223 participants revealed that nearly half of the ­participants (*n* = 104, 46.6%) reported medication-related issues. Other documented concerns included discussions about diabetes testing supplies (40.4%), nutrition concerns (10.8%), mood problems (8.1%), and clinic eligibility (4.5%). The CHW–participant conversation data for most non-medication-related issues were limited by documentation methodology. For example, because validated assessment tools (eg, PHQ-9, GAD-7) were not part of the study protocols, it is unknown how often a participant may have experienced unreported mood symptoms or whether comments about feeling “depressed” or “anxious” were colloquial or indicative of clinical anxiety or depression. Similarly, because diabetes testing supplies were provided to participants as part of some of the included studies, CHW documentation of testing supply provision may have been proactive, rather than a response to a participant’s concern.

We reviewed the data on the most common concern, medication issues, using cut-off points rather than continuous outcomes due to the absence of pre/post data, unlike with HbA1c. This method also facilitated interpretation and allowed for the categorization of data into meaningful groups to aid practical decision-making. Older participants were more likely to report medication-related issues during the study. Among those aged 50 years and older, 51.9% (81 out of 156) reported medication issues, compared to 34.3% (23 out of 67) of participants under 50 years, χ^2^(1) = 5.83, *p* = .016. Likewise, 73.7% (14 out of 19) of participants aged 65 years and older raised a medication-related concern with the CHW, compared to 44.1% (90 out of 204) of those 64 years or younger, χ^2^(1) = 6.11, *p* = .013.

## Discussion

To examine age-related variations in HbA1c changes, we conducted a retrospective analysis of 10 studies on CHW-led mHealth and group education diabetes interventions for Hispanic patients. HbA1c improved across all age groups, with the greatest reduction observed in middle-aged participants, while reductions plateaued in older adults. Older adults also reported more medication-related issues compared to younger adults. The findings in this study are important, as they highlight the need for tailored interventions to address the unique challenges faced by older adults in diabetes management.

As projections forecast that adults aged 65 years and older will outnumber children under 18 years within a decade in the United States and within 50 years globally,^35^^,^^36^ it is vital to identify effective approaches to diabetes management in older adults. Large, rigorous RCTs of type 2 diabetes medical ­management have not shown an age effect on HbA1c change resulting from medication interventions.^37^ However, age effects from adjunct interventions supporting lifestyle and behavioral aspects of diabetes management have been less rigorously ­studied. In addition, RCTs may fail to capture the real-world challenges of diabetes management experienced by older adults, including multimorbidity, polypharmacy, social isolation, and frailty, as well as socio-economic barriers such as transportation and financial constraints faced by low-income populations.^38-41^

A study in Korea found differences in patient-reported and clinical characteristics by decade, suggesting that diabetes self-management education may need age-specific tailoring, similar to culturally tailored programs.^42^ Another study on telehealth-based diabetes education found that older adults (>55 years) required more time for education than younger adults.^43^ A systematic review of telemedicine and CHW interventions for adults >50 years found HbA1c reduction effectiveness but lacked data on adults >65 years and comparisons with younger groups.^44^ Additionally, a small randomized trial involving CHW-led interventions showed greater HbA1c reductions in adults over 55, but the sample size for those >65 years was unclear.^45^

The age-related variation in HbA1c reduction observed in our study warrants further investigation. The smaller HbA1c reduction in older adults could reflect the less-stringent glycemic targets recommended by the American Diabetes Association to minimize hypoglycemia, which would suggest that the intervention’s effect was not diminished. However, we observed that older adults encountered age-specific challenges not addressed by a program designed for the general adult population, such as cognitive impairment, social isolation, or transportation barriers. Additionally, older adults were more likely to report medication-related issues, raising the question of whether these issues contributed to the reduced HbA1c improvement in this group. Tighter glycemic control in older adults is associated with a heightened risk of severe hypoglycemia.^46^ Common diabetes medications, including sliding-scale insulin, sulfonylureas, and SGLT2 inhibitors, are considered potentially inappropriate for older adults due to the risk of hypoglycemia and other adverse effects, which may impact adherence.^47^ Given that medication side effects are a known barrier to adherence and can significantly affect diabetes control, the higher rate of medication issues in older adults may have contributed to the smaller HbA1c reductions observed in this group.

An emerging approach to improving care for older adults is the 4M Framework (*What Matters, Medication, Mentation, and Mobility*) developed by the Institute for Healthcare Improvement.^48^ The 4Ms have demonstrated reduced functional decline and healthcare costs; however, challenges in identifying appropriate personnel to implement these interventions have limited broader adoption.^48-50^ Though still in its infancy, there is growing interest in involving CHWs in the 4Ms.^49^^,^^50^ The trusted relationship between CHWs and patients positions them uniquely to address the 4Ms. While not a primary focus of our studies, we observed that CHW–participant conversations naturally encompassed discussions on what matters to the patient and medication concerns (eg, adherence, refills) and could be easily adapted to incorporate mobility and mentation assessments. Further research is needed to explore the potential impact of CHWs in managing these areas, particularly among older Hispanic adults with diabetes.

A key strength of this study is its comprehensive analysis of 9 years of data from 301 Hispanic individuals with or at risk for type 2 diabetes, providing valuable insights into the long-term trends of HbA1c reduction across different age groups. Additional strengths include the inclusion of multiple prospective studies across several health systems, all examining the impact of a CHW-led mHealth intervention for Hispanics with or at risk for diabetes. Limitations of this study include its retrospective design and the limited number of participants on both ends of the age spectrum. While the very old were not well represented in this study, more than 2-thirds of ­participants (69.6%) were aged at least 50 years, and 21.6% were greater than 60 years. The study’s focus on Hispanic patients without insurance limits its generalizability to other populations.

Future research is needed to explore the impact of CHW interventions on older adults with diabetes, particularly considering factors such as multimorbidity and frailty, and how these interventions may need to be adapted for this population. Studies should also incorporate data on the 4Ms in the context of CHW-led interventions.

## Conclusions

We found that CHW-led diabetes interventions produced varying effects on HbA1c across the age spectrum, with the most significant reductions in individuals under 50 and minimal impact in older adults. Future research should explore the factors driving these age-related differences and develop targeted interventions, such as integrating CHW-led 4Ms initiatives into diabetes programs, to better address the unique needs of older adults and improve diabetes management.

## Supplementary Material

igaf121_Supplementary_Data

## Data Availability

The data supporting the findings of this study are available from the corresponding author upon reasonable request. The NIH studies were preregistered: NCT03394456, NCT04835493.
