# Supplementary material for: Age-related variations in HbA1c improvements: insights from a telehealth-supported community-based intervention
Source: Innov Aging. 2025 Oct 30;9(11):igaf121. doi: 10.1093/geroni/igaf121 (PMC12640246; doi:10.1093/geroni/igaf121)
Supplement: igaf121_Supplementary_Data [file igaf121_supplementary_data.zip › innage suppl Porterfield et al.docx]

***Innovation in Aging* Supplementary Material: Porterfield et al. Age-Related Variations in HbA1c Improvements: Insights from a Telehealth-Supported Community-Based Intervention.**

**Supplementary Table 1.** Estimated age-specific HbA1c changes from baseline to 6-months.

| **AGE** | **Estimated change** | **Lower 95% CI** | **Upper 95% CI** | **Stepdown Bonferroni** ***p*-value** |
| --- | --- | --- | --- | --- |
| **40** | -0.8330 | -1.3282 | -0.3378 | 0.0126 |
| **41** | -0.8959 | -1.3409 | -0.4508 | 0.0014 |
| **42** | -0.9569 | -1.3623 | -0.5515 | 0.0001 |
| **43** | -1.0142 | -1.3921 | -0.6364 | <0.0001 |
| **44** | -1.0660 | -1.4283 | -0.7038 | <0.0001 |
| **45** | -1.1105 | -1.4664 | -0.7546 | <0.0001 |
| **46** | -1.1457 | -1.5002 | -0.7913 | <0.0001 |
| **47** | -1.1699 | -1.5230 | -0.8168 | <0.0001 |
| **48** | -1.1812 | -1.5287 | -0.8336 | <0.0001 |
| **49** | -1.1777 | -1.5125 | -0.8430 | <0.0001 |
| **50** | -1.1577 | -1.4713 | -0.8441 | <0.0001 |
| **51** | -1.1204 | -1.4078 | -0.8330 | <0.0001 |
| **52** | -1.0700 | -1.3365 | -0.8034 | <0.0001 |
| **53** | -1.0117 | -1.2729 | -0.7504 | <0.0001 |
| **54** | -0.9507 | -1.2241 | -0.6774 | <0.0001 |
| **55** | -0.8925 | -1.1875 | -0.5975 | <0.0001 |
| **56** | -0.8411 | -1.1562 | -0.5261 | <0.0001 |
| **57** | -0.7969 | -1.1252 | -0.4687 | 0.0001 |
| **58** | -0.7591 | -1.0930 | -0.4252 | 0.0002 |
| **59** | -0.7268 | -1.0602 | -0.3934 | 0.0005 |
| **60** | -0.6992 | -1.0285 | -0.3700 | 0.0007 |
| **61** | -0.6757 | -1.0006 | -0.3507 | 0.0010 |
| **62** | -0.6552 | -0.9793 | -0.3312 | 0.0014 |
| **63** | -0.6372 | -0.9674 | -0.3070 | 0.0025 |
| **64** | -0.6208 | -0.9663 | -0.2753 | 0.0062 |
| **65** | -0.6051 | -0.9755 | -0.2348 | 0.0160 |
| **66** | -0.5896 | -0.9931 | -0.1861 | 0.0433 |
| **67** | -0.5741 | -1.0171 | -0.1311 | 0.1015 |
| **68** | -0.5586 | -1.0460 | -0.0711 | 0.1989 |
| **69** | -0.5431 | -1.0786 | -0.0075 | 0.3282 |
| **70** | -0.5276 | -1.1139 | 0.0588 | 0.4660 |
| **71** | -0.5120 | -1.1514 | 0.1273 | 0.5802 |
| **72** | -0.4965 | -1.1905 | 0.1974 | 0.6404 |
| **73** | -0.4810 | -1.2308 | 0.2688 | 0.6404 |
| **74** | -0.4655 | -1.2721 | 0.3412 | 0.6404 |
| **75** | -0.4500 | -1.3143 | 0.4143 | 0.6404 |

**Supplementary Table 2**. Estimated age-specific HbA1c changes from baseline to participant study endpoint (6-, 12-, 15-months).

| **AGE** | **Estimated change** | **Lower 95% CI** | **Upper 95% CI** | **Stepdown Bonferroni *p*-value** |
| --- | --- | --- | --- | --- |
| **40** | -0.6668 | -1.1456 | -0.1880 | 0.0651 |
| **41** | -0.7159 | -1.1511 | -0.2806 | 0.0162 |
| **42** | -0.7636 | -1.1655 | -0.3616 | 0.0036 |
| **43** | -0.8086 | -1.1886 | -0.4287 | 0.0009 |
| **44** | -0.8497 | -1.2181 | -0.4813 | 0.0002 |
| **45** | -0.8854 | -1.2498 | -0.5210 | 0.0001 |
| **46** | -0.9144 | -1.2782 | -0.5507 | <0.0001 |
| **47** | -0.9355 | -1.2976 | -0.5733 | <0.0001 |
| **48** | -0.9472 | -1.3029 | -0.5914 | <0.0001 |
| **49** | -0.9482 | -1.2900 | -0.6063 | <0.0001 |
| **50** | -0.9372 | -1.2569 | -0.6175 | <0.0001 |
| **51** | -0.9137 | -1.2064 | -0.6210 | <0.0001 |
| **52** | -0.8808 | -1.1521 | -0.6096 | <0.0001 |
| **53** | -0.8424 | -1.1078 | -0.5770 | <0.0001 |
| **54** | -0.8024 | -1.0795 | -0.5253 | <0.0001 |
| **55** | -0.7646 | -1.0633 | -0.4659 | <0.0001 |
| **56** | -0.7323 | -1.0514 | -0.4132 | 0.0002 |
| **57** | -0.7056 | -1.0387 | -0.3725 | 0.0009 |
| **58** | -0.6839 | -1.0237 | -0.3441 | 0.0020 |
| **59** | -0.6665 | -1.0069 | -0.3261 | 0.0029 |
| **60** | -0.6528 | -0.9900 | -0.3156 | 0.0033 |
| **61** | -0.6422 | -0.9756 | -0.3088 | 0.0033 |
| **62** | -0.6341 | -0.9666 | -0.3016 | 0.0036 |
| **63** | -0.6279 | -0.9655 | -0.2902 | 0.0045 |
| **64** | -0.6229 | -0.9739 | -0.2718 | 0.0078 |
| **65** | -0.6185 | -0.9918 | -0.2451 | 0.0162 |
| **66** | -0.6142 | -1.0178 | -0.2106 | 0.0328 |
| **67** | -0.6099 | -1.0501 | -0.1698 | 0.0651 |
| **68** | -0.6057 | -1.0873 | -0.1240 | 0.1112 |
| **69** | -0.6014 | -1.1283 | -0.0746 | 0.1779 |
| **70** | -0.5971 | -1.1721 | -0.0222 | 0.2511 |
| **71** | -0.5929 | -1.2181 | 0.0324 | 0.3150 |
| **72** | -0.5886 | -1.2658 | 0.0886 | 0.3529 |
| **73** | -0.5843 | -1.3148 | 0.1462 | 0.3529 |
| **74** | -0.5800 | -1.3650 | 0.2049 | 0.3529 |
| **75** | -0.5758 | -1.4160 | 0.2645 | 0.3529 |
